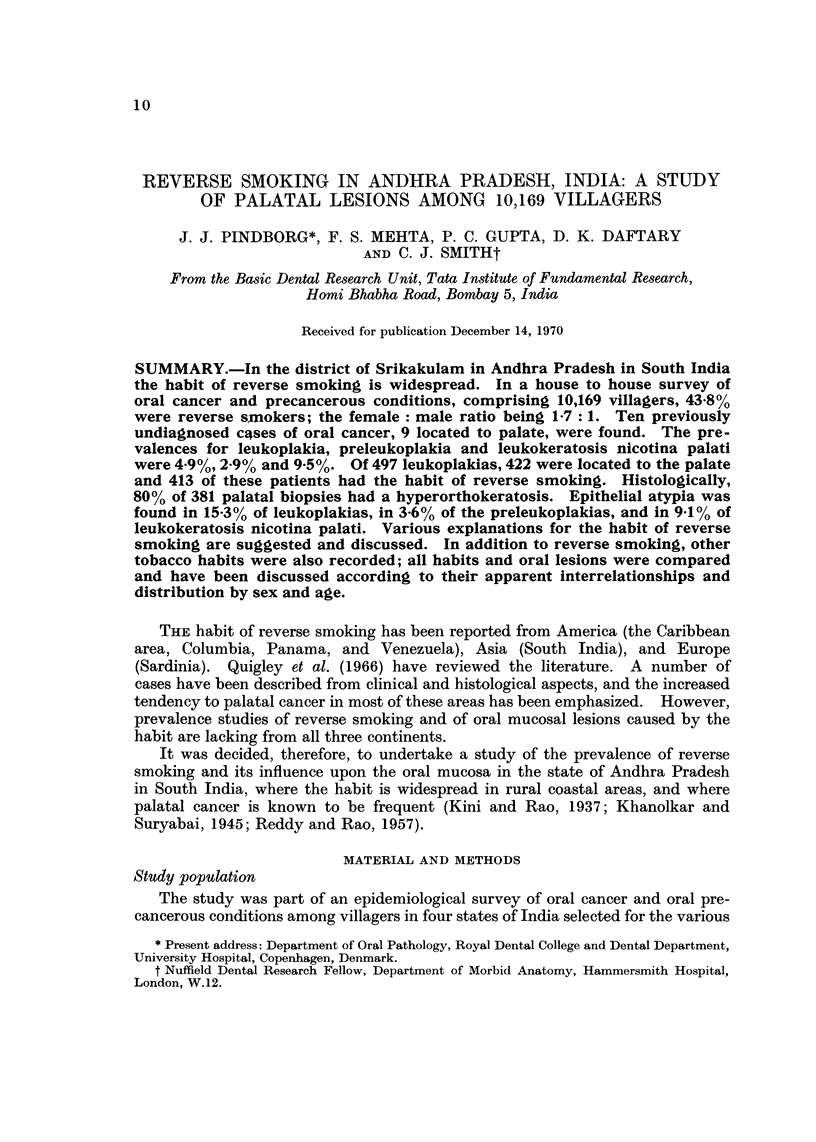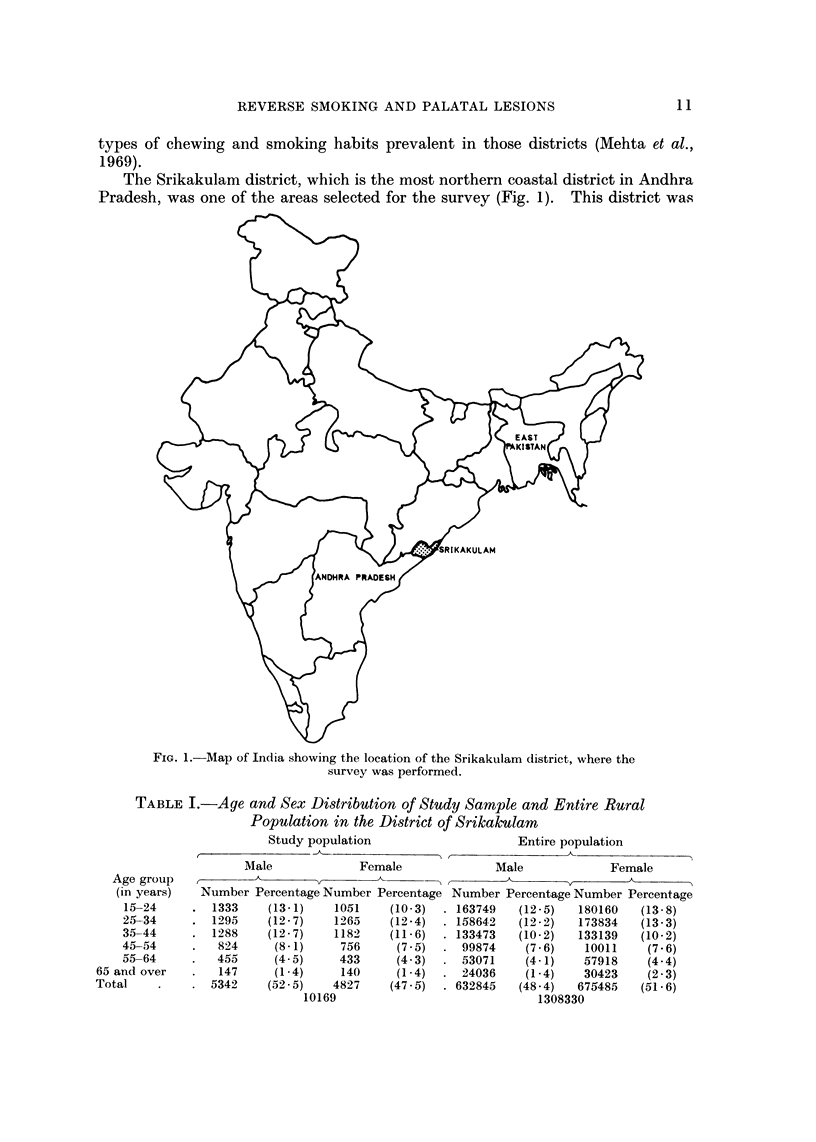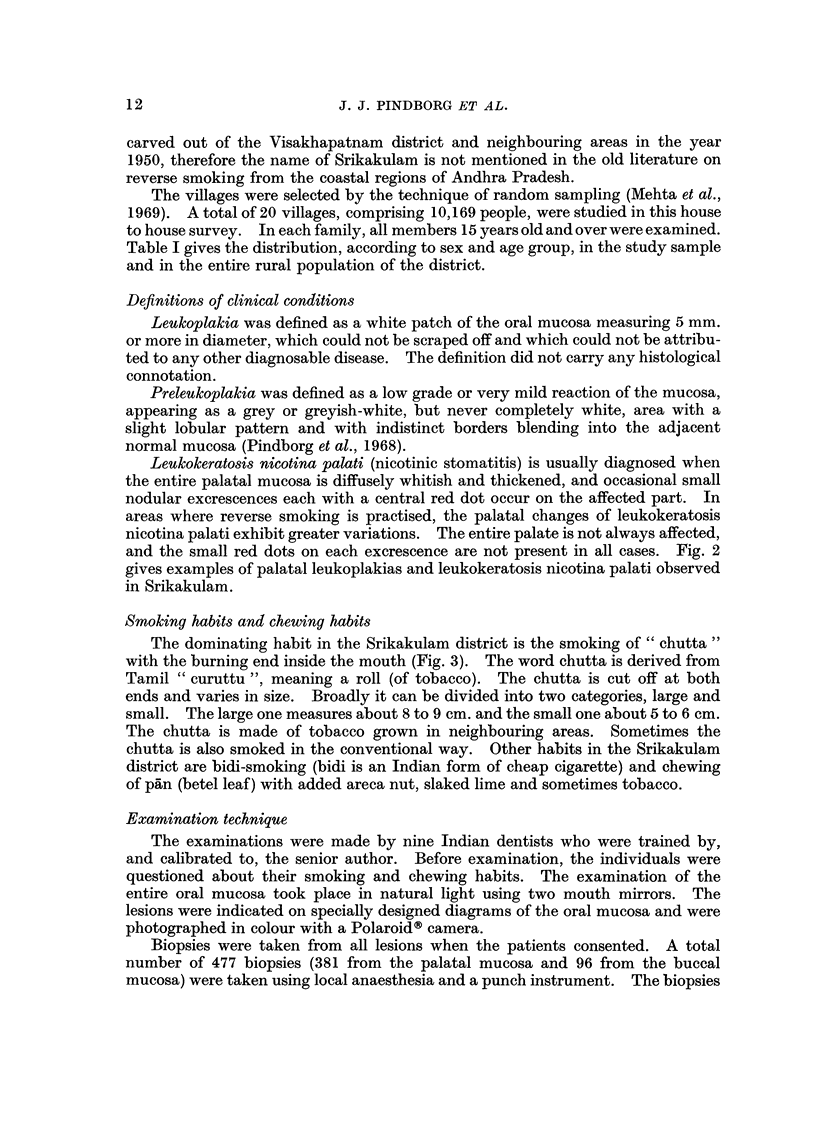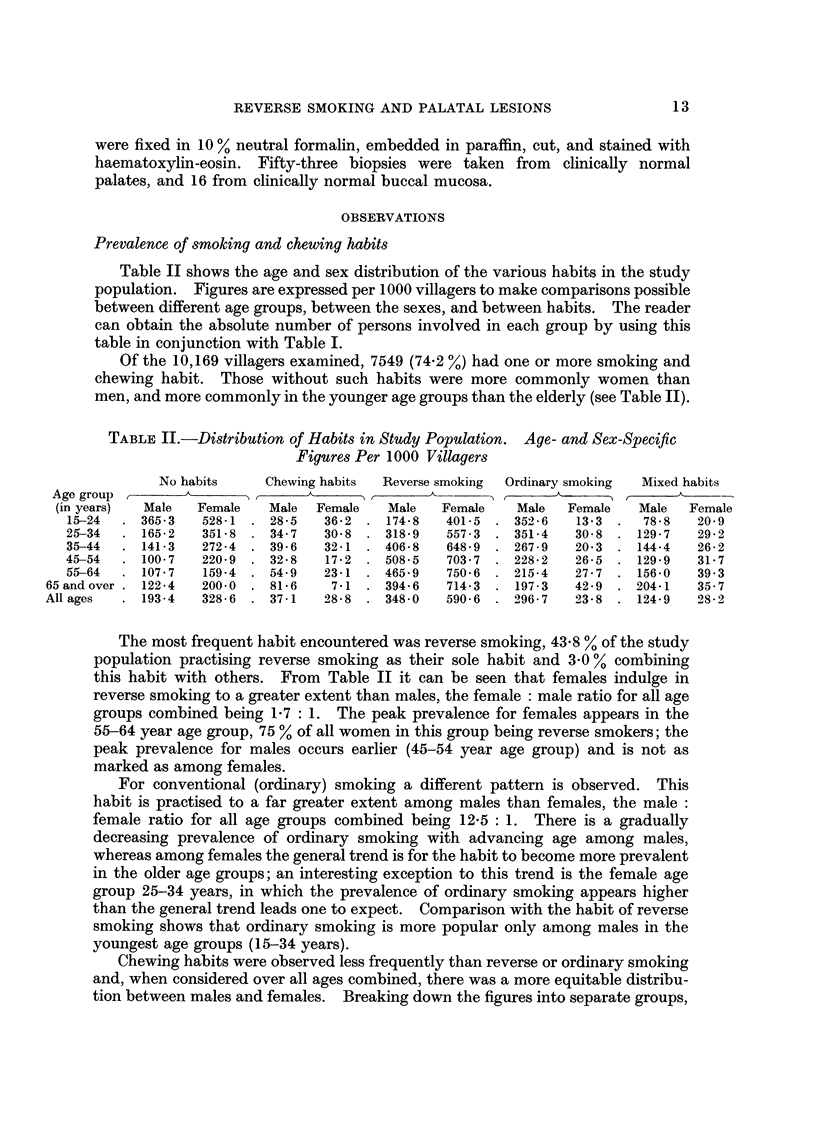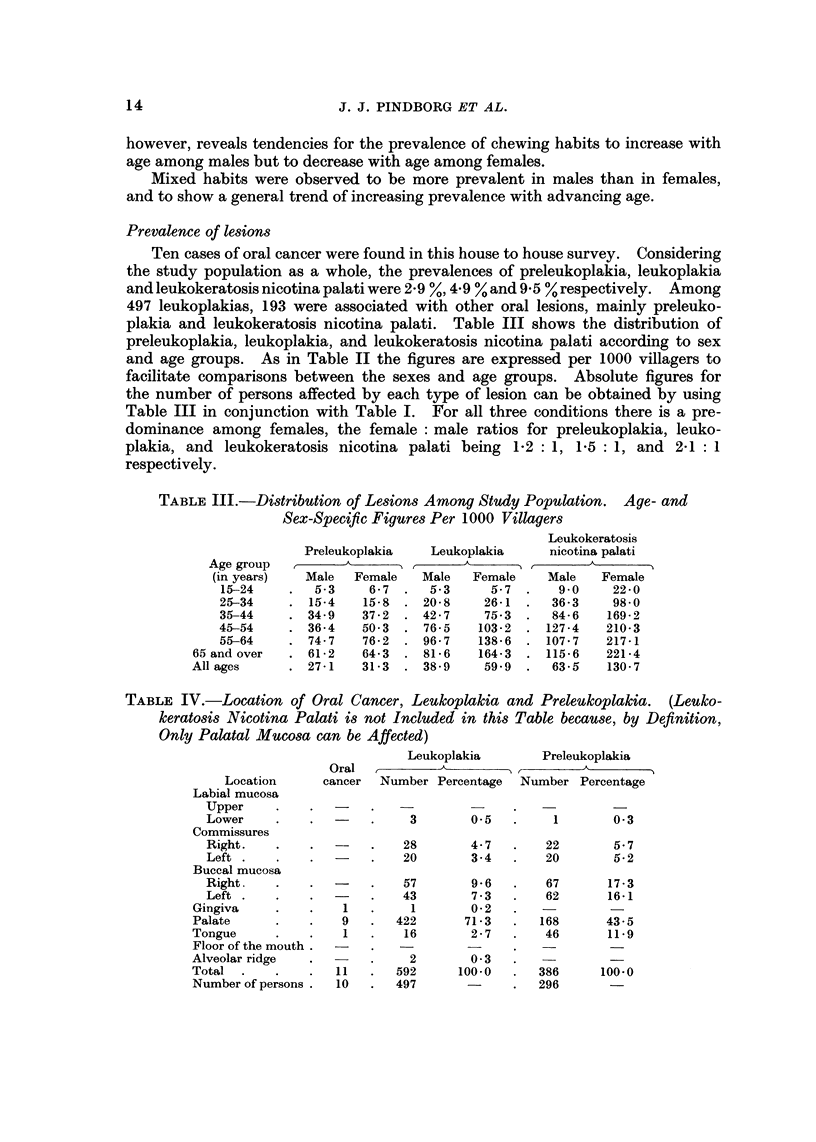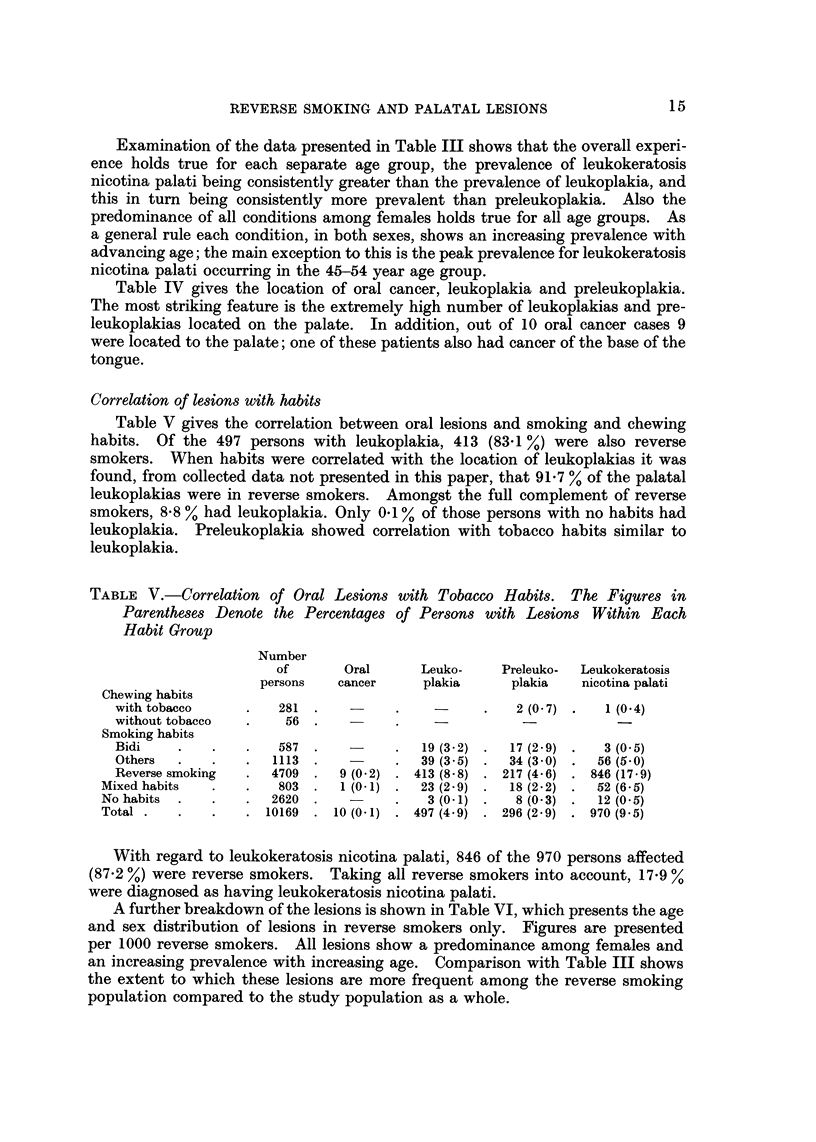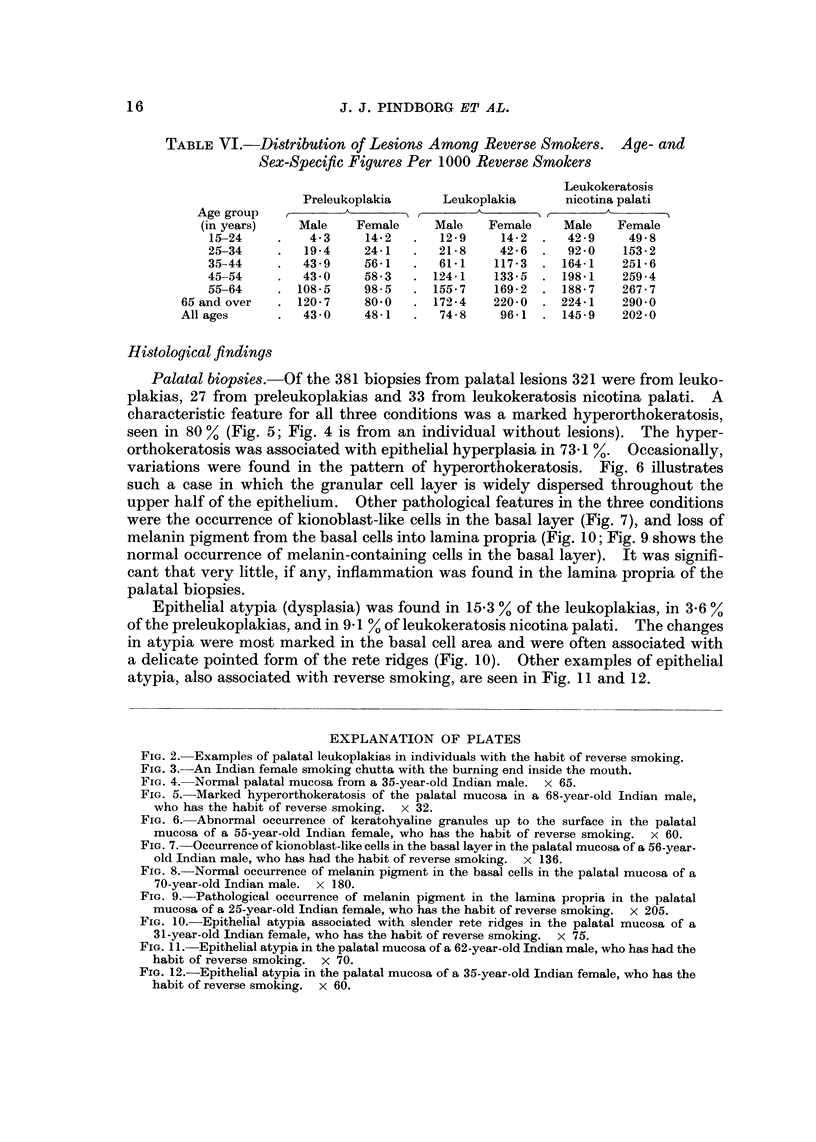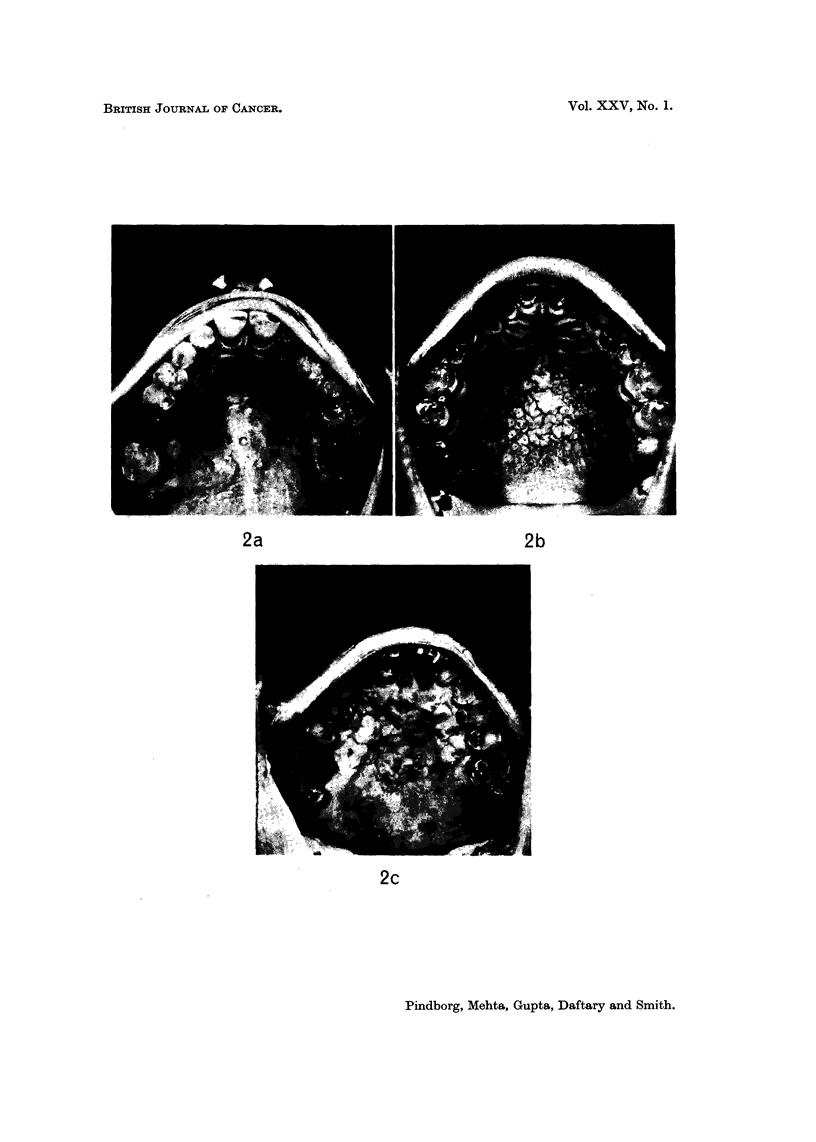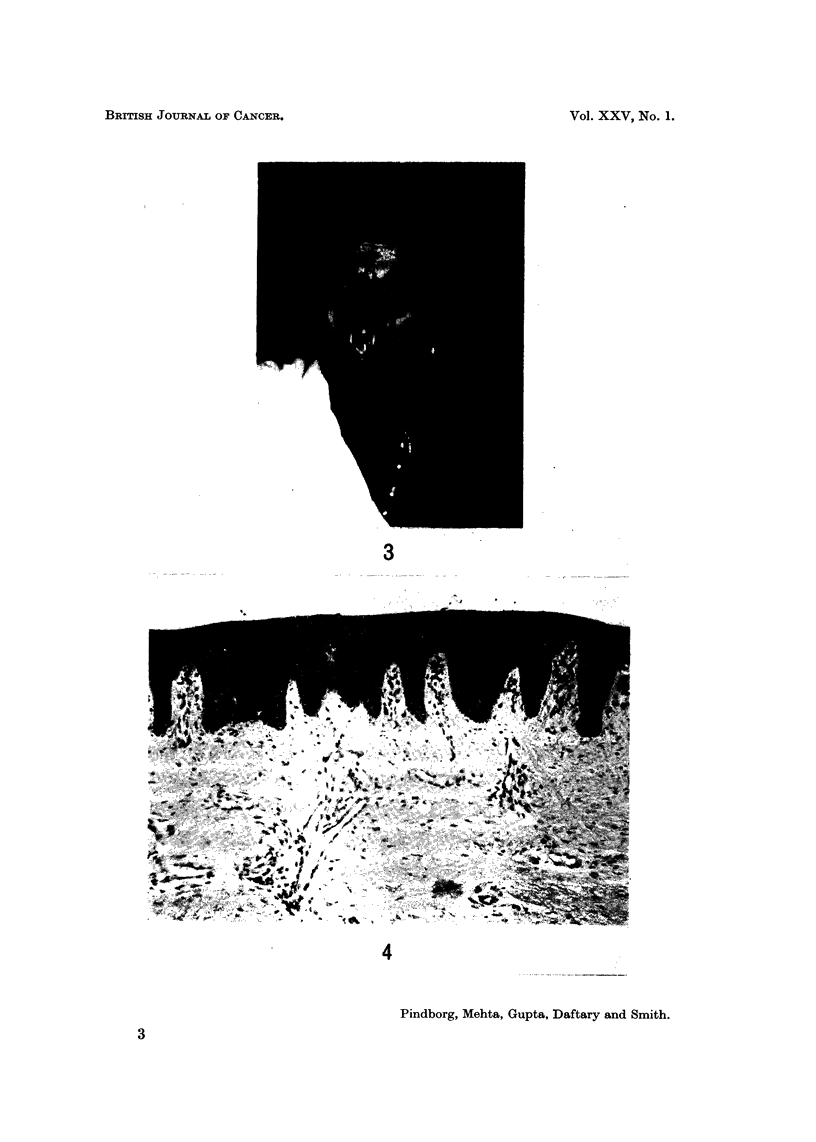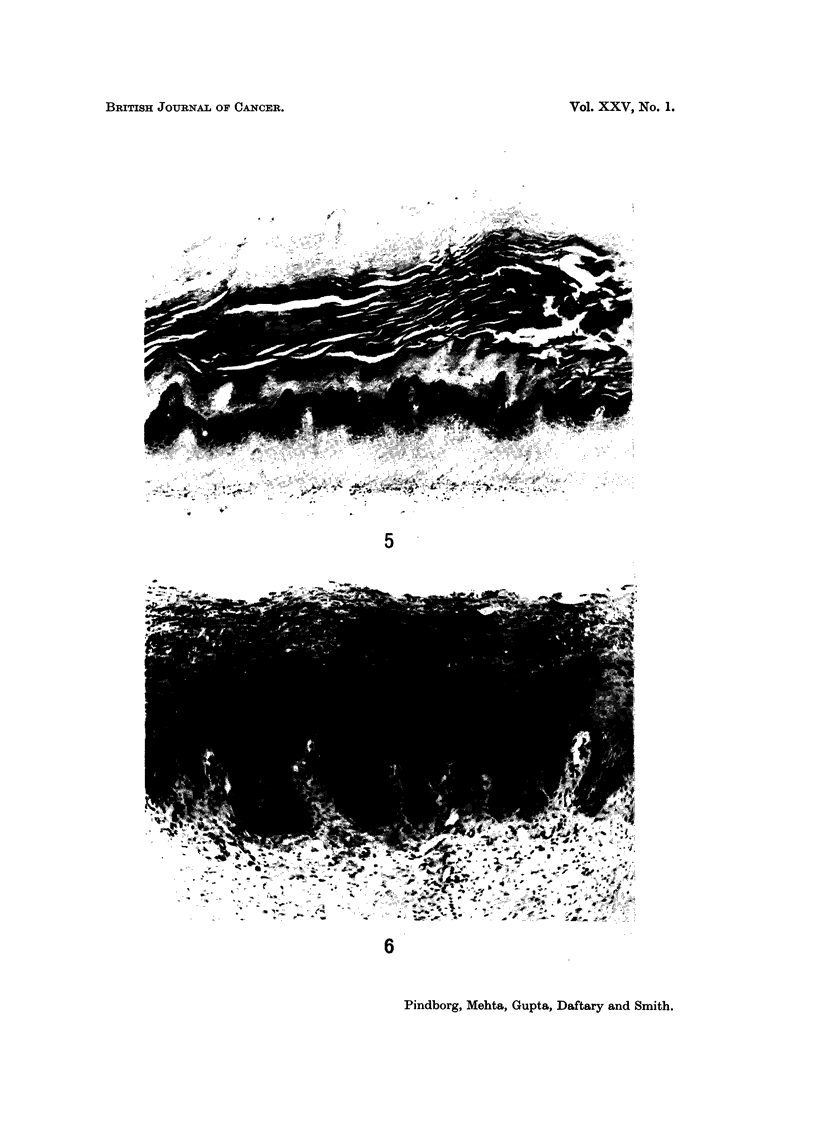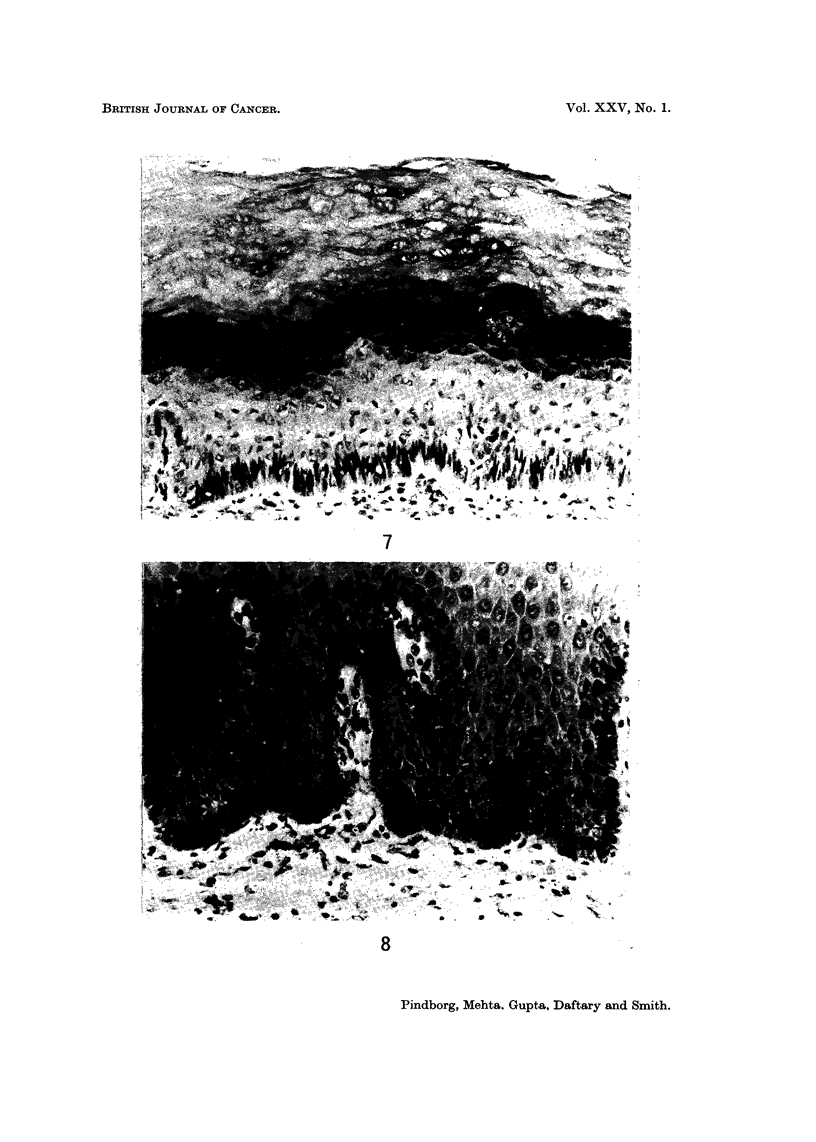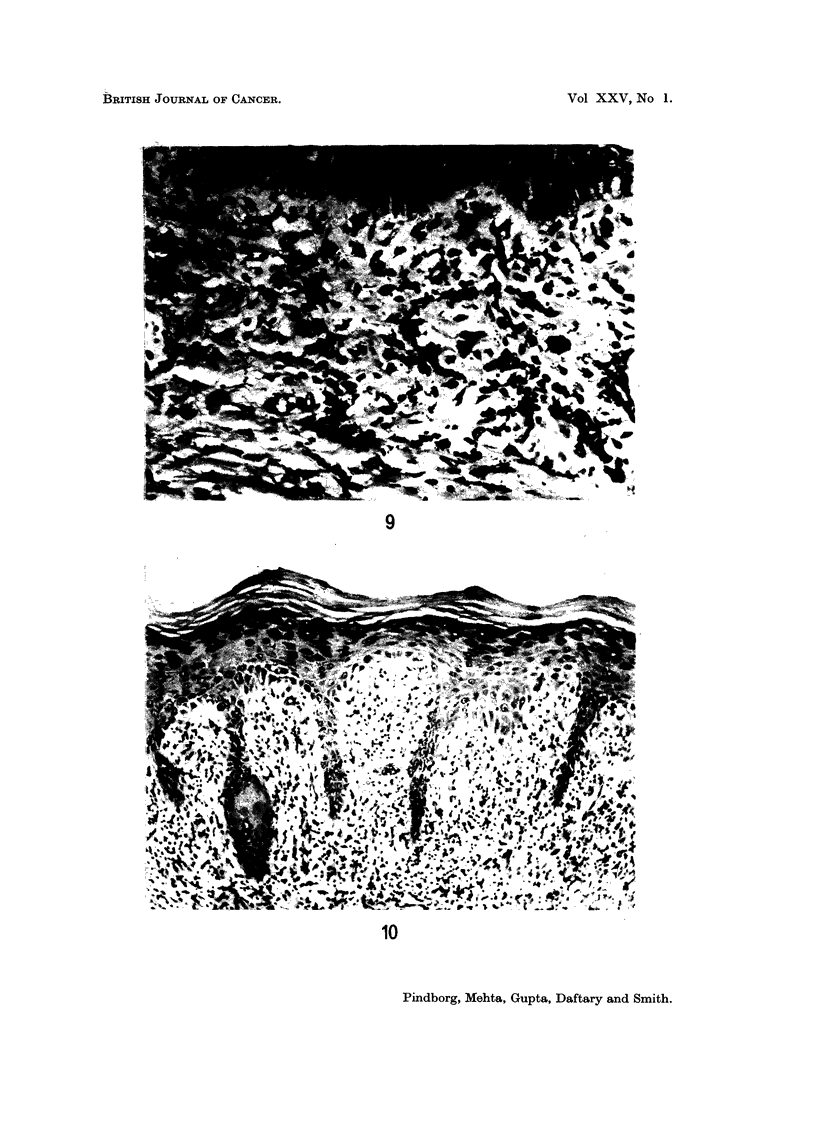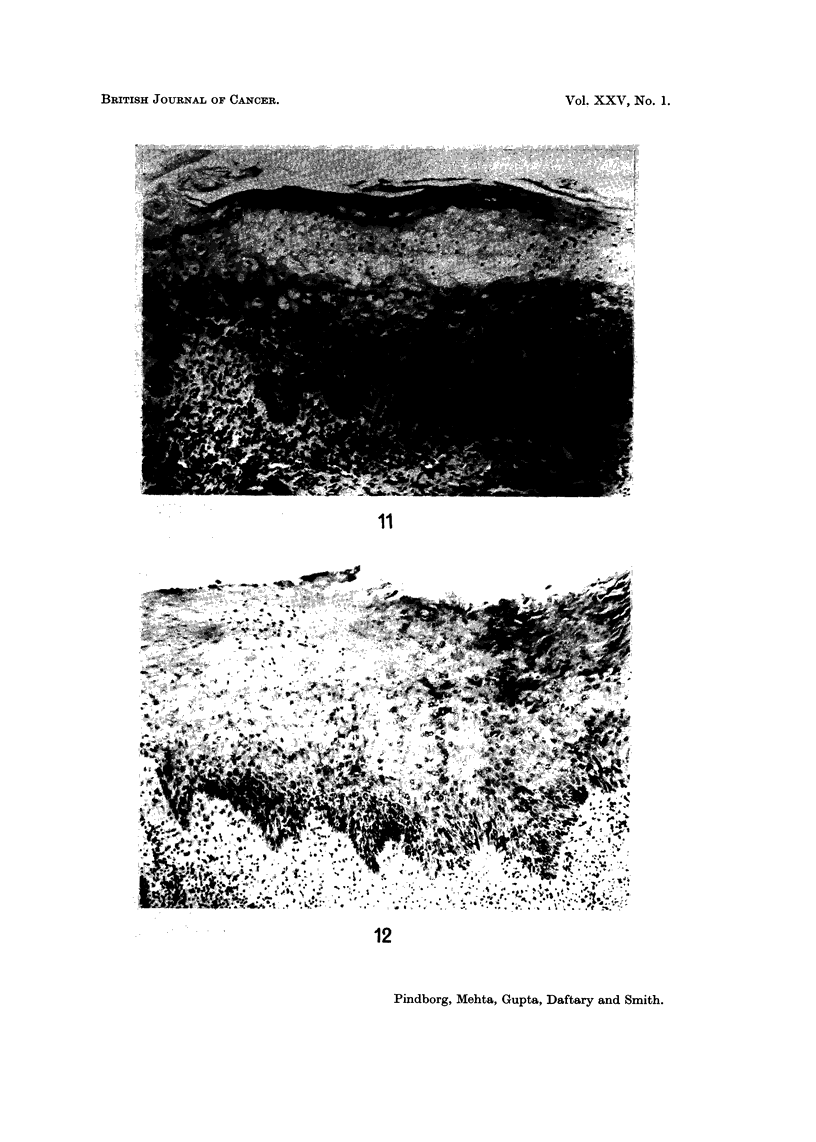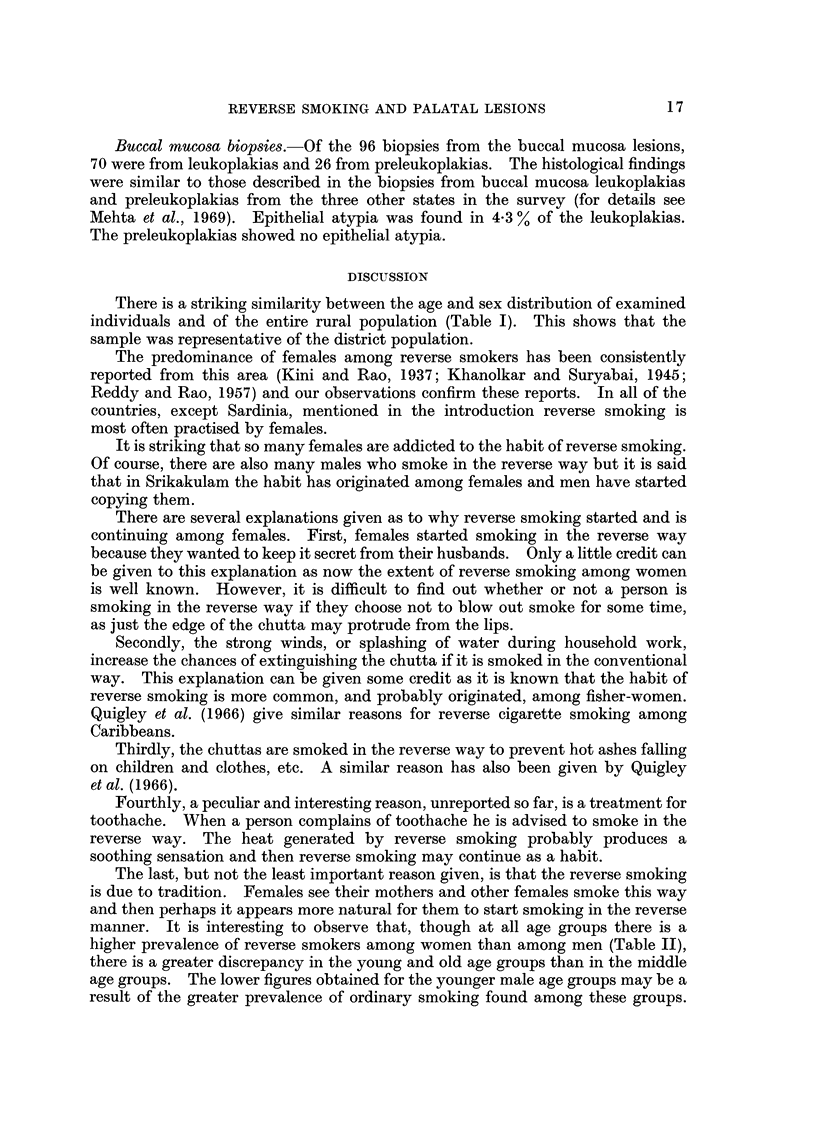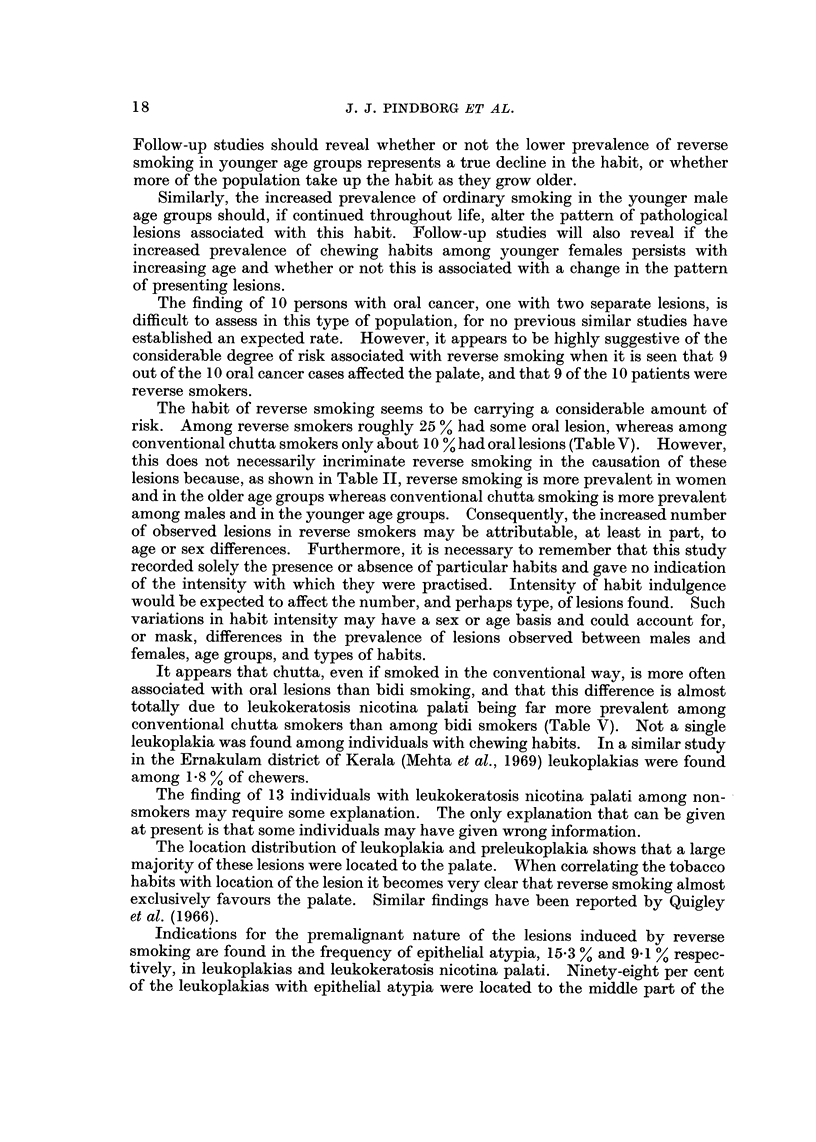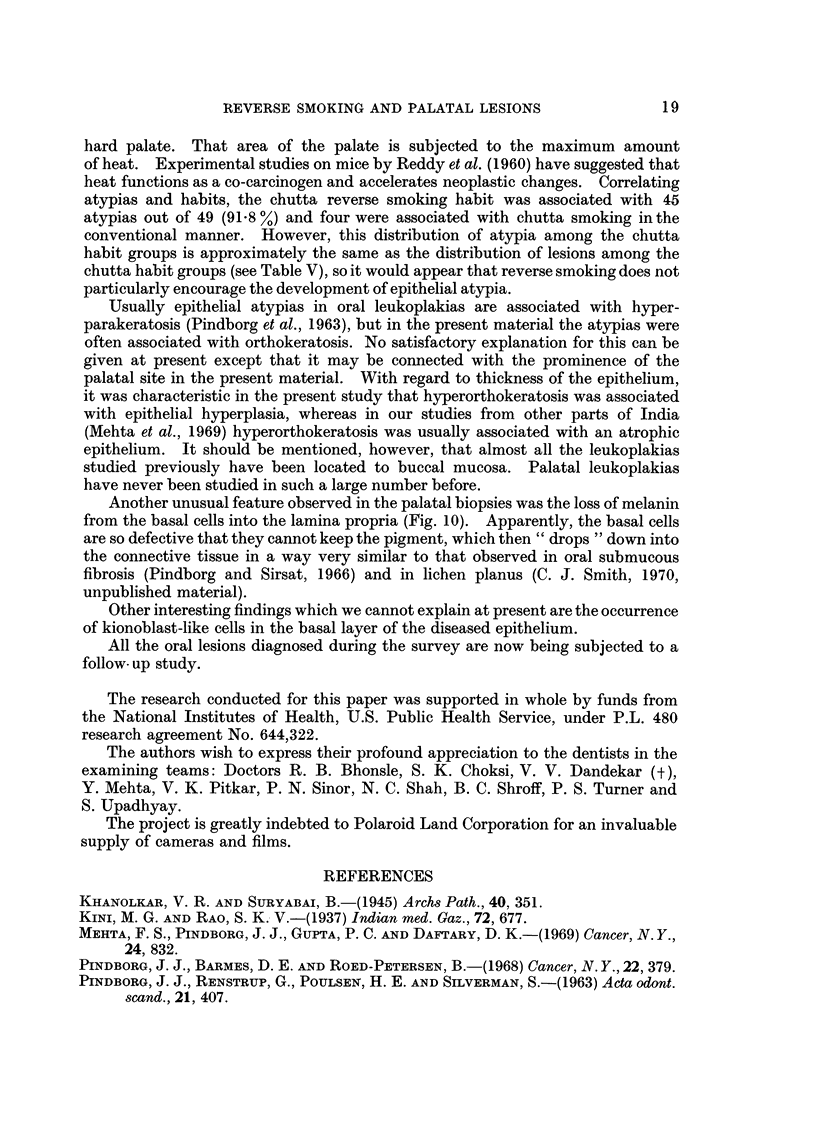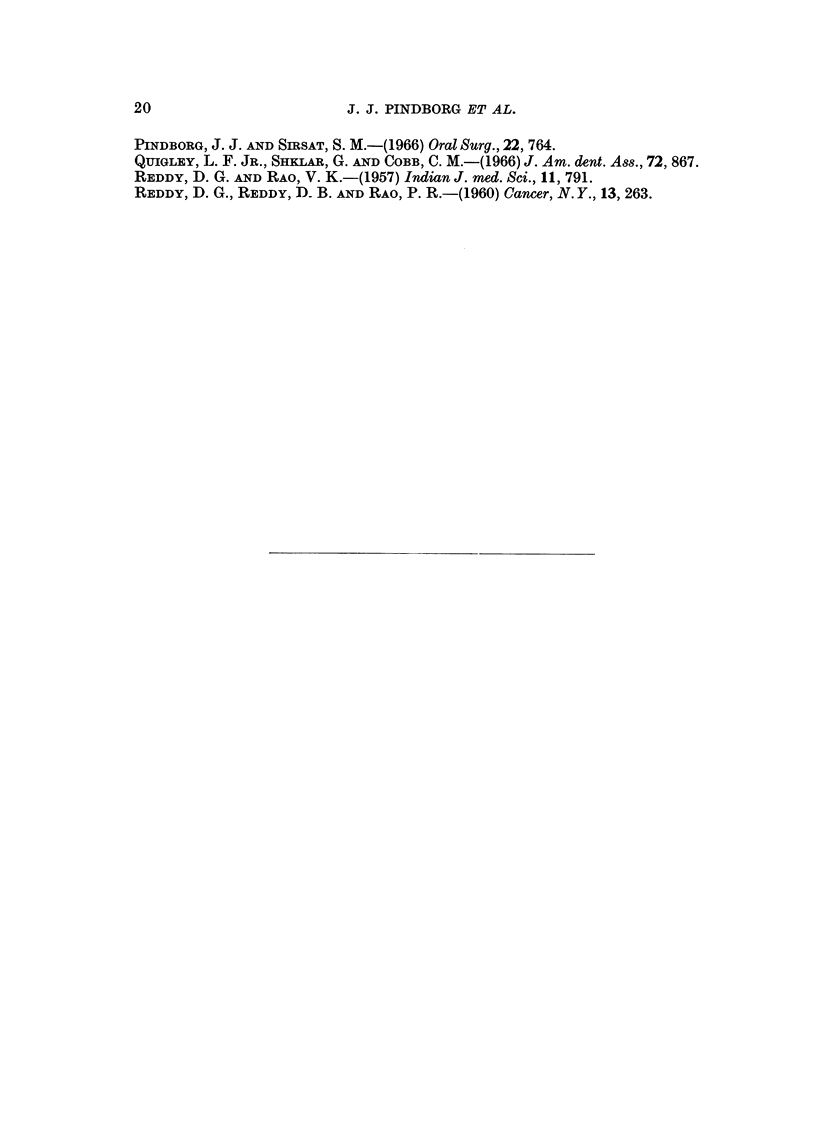# Reverse Smoking in Andhra Pradesh, India: A Study of Palatal Lesions among 10,169 Villagers

**DOI:** 10.1038/bjc.1971.2

**Published:** 1971-03

**Authors:** J. J. Pindborg, F. S. Mehta, P. C. Gupta, D. K. Daftary, C. J. Smith

## Abstract

**Images:**


### REVERSE SMOKING IN ANDHRA PRADESH, INDIA: A STUDY OF PALATAL LESIONS AMONG 10,169 VILLAGERS

J. J. PINDBORG*, F. S. MEHTA, P. C. GUPTA, D. K. DAFTARY AND C. J. SMITH†

*From the Basic Dental Research Unit, Tata Institute of Fundamental Research, Homi Bhabha Road, Bombay 5, India*

Received for publication December 14, 1970

**SUMMARY.—In the district of Srikakulam in Andhra Pradesh in South India the habit of reverse smoking is widespread. In a house to house survey of oral cancer and precancerous conditions, comprising 10,169 villagers, 43·8% were reverse smokers; the female : male ratio being 1·7 : 1. Ten previously undiagnosed cases of oral cancer, 9 located to palate, were found. The prevalences for leukoplakia, preleukoplakia and leukokeratosis nicotina palati were 4·9%, 2·9% and 9·5%. Of 497 leukoplakias, 422 were located to the palate and 413 of these patients had the habit of reverse smoking. Histologically, 80% of 381 palatal biopsies had a hyperorthokeratosis. Epithelial atypia was found in 15·3% of leukoplakias, in 3·6% of the preleukoplakias, and in 9·1% of leukokeratosis nicotina palati. Various explanations for the habit of reverse smoking are suggested and discussed. In addition to reverse smoking, other tobacco habits were also recorded; all habits and oral lesions were compared and have been discussed according to their apparent interrelationships and distribution by sex and age.**

THE habit of reverse smoking has been reported from America (the Caribbean area, Columbia, Panama, and Venezuela), Asia (South India), and Europe (Sardinia). Quigley *et al.* (1966) have reviewed the literature. A number of cases have been described from clinical and histological aspects, and the increased tendency to palatal cancer in most of these areas has been emphasized. However, prevalence studies of reverse smoking and of oral mucosal lesions caused by the habit are lacking from all three continents.

It was decided, therefore, to undertake a study of the prevalence of reverse smoking and its influence upon the oral mucosa in the state of Andhra Pradesh in South India, where the habit is widespread in rural coastal areas, and where palatal cancer is known to be frequent (Kini and Rao, 1937; Khanolkar and Suryabai, 1945; Reddy and Rao, 1957).

### MATERIAL AND METHODS

### *Study population*

The study was part of an epidemiological survey of oral cancer and oral precancerous conditions among villagers in four states of India selected for the various

* Present address: Department of Oral Pathology, Royal Dental College and Dental Department, University Hospital, Copenhagen, Denmark.

† Nuffield Dental Research Fellow, Department of Morbid Anatomy, Hammersmith Hospital, London, W.12.

types of chewing and smoking habits prevalent in those districts (Mehta *et al.*, 1969).

The Srikakulam district, which is the most northern coastal district in Andhra Pradesh, was one of the areas selected for the survey (Fig. 1). This district was

FIG. 1.—Map of India showing the location of the Srikakulam district, where the survey was performed.

TABLE I.—*Age and Sex Distribution of Study Sample and Entire Rural Population in the District of Srikakulam*

| Age group (in years) | Study population | | | | Entire population | | | |
|---|---|---|---|---|---|---|---|---|
| | Male | | Female | | Male | | Female | |
| | Number | Percentage | Number | Percentage | Number | Percentage | Number | Percentage |
| 15–24 | 1333 | (13·1) | 1051 | (10·3) | 163749 | (12·5) | 180160 | (13·8) |
| 25–34 | 1295 | (12·7) | 1265 | (12·4) | 158642 | (12·2) | 173834 | (13·3) |
| 35–44 | 1288 | (12·7) | 1182 | (11·6) | 133473 | (10·2) | 133139 | (10·2) |
| 45–54 | 824 | (8·1) | 756 | (7·5) | 99874 | (7·6) | 10011 | (7·6) |
| 55–64 | 455 | (4·5) | 433 | (4·3) | 53071 | (4·1) | 57918 | (4·4) |
| 65 and over | 147 | (1·4) | 140 | (1·4) | 24036 | (1·4) | 30423 | (2·3) |
| Total | 5342 | (52·5) | 4827 | (47·5) | 632845 | (48·4) | 675485 | (51·6) |
| | 10169 | | | | 1308330 | | | |

carved out of the Visakhapatnam district and neighbouring areas in the year 1950, therefore the name of Srikakulam is not mentioned in the old literature on reverse smoking from the coastal regions of Andhra Pradesh.

The villages were selected by the technique of random sampling (Mehta *et al.*, 1969). A total of 20 villages, comprising 10,169 people, were studied in this house to house survey. In each family, all members 15 years old and over were examined. Table I gives the distribution, according to sex and age group, in the study sample and in the entire rural population of the district.

*Definitions of clinical conditions*

*Leukoplakia* was defined as a white patch of the oral mucosa measuring 5 mm. or more in diameter, which could not be scraped off and which could not be attributed to any other diagnosable disease. The definition did not carry any histological connotation.

*Preleukoplakia* was defined as a low grade or very mild reaction of the mucosa, appearing as a grey or greyish-white, but never completely white, area with a slight lobular pattern and with indistinct borders blending into the adjacent normal mucosa (Pindborg *et al.*, 1968).

*Leukokeratosis nicotina palati* (nicotinic stomatitis) is usually diagnosed when the entire palatal mucosa is diffusely whitish and thickened, and occasional small nodular excrescences each with a central red dot occur on the affected part. In areas where reverse smoking is practised, the palatal changes of leukokeratosis nicotina palati exhibit greater variations. The entire palate is not always affected, and the small red dots on each excrescence are not present in all cases. Fig. 2 gives examples of palatal leukoplakias and leukokeratosis nicotina palati observed in Srikakulam.

*Smoking habits and chewing habits*

The dominating habit in the Srikakulam district is the smoking of " chutta " with the burning end inside the mouth (Fig. 3). The word chutta is derived from Tamil " curuttu ", meaning a roll (of tobacco). The chutta is cut off at both ends and varies in size. Broadly it can be divided into two categories, large and small. The large one measures about 8 to 9 cm. and the small one about 5 to 6 cm. The chutta is made of tobacco grown in neighbouring areas. Sometimes the chutta is also smoked in the conventional way. Other habits in the Srikakulam district are bidi-smoking (bidi is an Indian form of cheap cigarette) and chewing of pān (betel leaf) with added areca nut, slaked lime and sometimes tobacco.

*Examination technique*

The examinations were made by nine Indian dentists who were trained by, and calibrated to, the senior author. Before examination, the individuals were questioned about their smoking and chewing habits. The examination of the entire oral mucosa took place in natural light using two mouth mirrors. The lesions were indicated on specially designed diagrams of the oral mucosa and were photographed in colour with a Polaroid® camera.

Biopsies were taken from all lesions when the patients consented. A total number of 477 biopsies (381 from the palatal mucosa and 96 from the buccal mucosa) were taken using local anaesthesia and a punch instrument. The biopsies were fixed in 10 % neutral formalin, embedded in paraffin, cut, and stained with haematoxylin-eosin. Fifty-three biopsies were taken from clinically normal palates, and 16 from clinically normal buccal mucosa.

### OBSERVATIONS

### *Prevalence of smoking and chewing habits*

Table II shows the age and sex distribution of the various habits in the study population. Figures are expressed per 1000 villagers to make comparisons possible between different age groups, between the sexes, and between habits. The reader can obtain the absolute number of persons involved in each group by using this table in conjunction with Table I.

Of the 10,169 villagers examined, 7549 (74·2 %) had one or more smoking and chewing habit. Those without such habits were more commonly women than men, and more commonly in the younger age groups than the elderly (see Table II).

TABLE II.—*Distribution of Habits in Study Population. Age- and Sex-Specific Figures Per* 1000 *Villagers*

| Age group (in years) | No habits | | Chewing habits | | Reverse smoking | | Ordinary smoking | | Mixed habits | |
|---|---|---|---|---|---|---|---|---|---|---|
| | Male | Female | Male | Female | Male | Female | Male | Female | Male | Female |
| 15–24 | 365·3 | 528·1 | 28·5 | 36·2 | 174·8 | 401·5 | 352·6 | 13·3 | 78·8 | 20·9 |
| 25–34 | 165·2 | 351·8 | 34·7 | 30·8 | 318·9 | 557·3 | 351·4 | 30·8 | 129·7 | 29·2 |
| 35–44 | 141·3 | 272·4 | 39·6 | 32·1 | 406·8 | 648·9 | 267·9 | 20·3 | 144·4 | 26·2 |
| 45–54 | 100·7 | 220·9 | 32·8 | 17·2 | 508·5 | 703·7 | 228·2 | 26·5 | 129·9 | 31·7 |
| 55–64 | 107·7 | 159·4 | 54·9 | 23·1 | 465·9 | 750·6 | 215·4 | 27·7 | 156·0 | 39·3 |
| 65 and over | 122·4 | 200·0 | 81·6 | 7·1 | 394·6 | 714·3 | 197·3 | 42·9 | 204·1 | 35·7 |
| All ages | 193·4 | 328·6 | 37·1 | 28·8 | 348·0 | 590·6 | 296·7 | 23·8 | 124·9 | 28·2 |

The most frequent habit encountered was reverse smoking, 43·8 % of the study population practising reverse smoking as their sole habit and 3·0 % combining this habit with others. From Table II it can be seen that females indulge in reverse smoking to a greater extent than males, the female : male ratio for all age groups combined being 1·7 : 1. The peak prevalence for females appears in the 55–64 year age group, 75 % of all women in this group being reverse smokers; the peak prevalence for males occurs earlier (45–54 year age group) and is not as marked as among females.

For conventional (ordinary) smoking a different pattern is observed. This habit is practised to a far greater extent among males than females, the male : female ratio for all age groups combined being 12·5 : 1. There is a gradually decreasing prevalence of ordinary smoking with advancing age among males, whereas among females the general trend is for the habit to become more prevalent in the older age groups; an interesting exception to this trend is the female age group 25–34 years, in which the prevalence of ordinary smoking appears higher than the general trend leads one to expect. Comparison with the habit of reverse smoking shows that ordinary smoking is more popular only among males in the youngest age groups (15–34 years).

Chewing habits were observed less frequently than reverse or ordinary smoking and, when considered over all ages combined, there was a more equitable distribution between males and females. Breaking down the figures into separate groups, however, reveals tendencies for the prevalence of chewing habits to increase with age among males but to decrease with age among females.

Mixed habits were observed to be more prevalent in males than in females, and to show a general trend of increasing prevalence with advancing age.

*Prevalence of lesions*

Ten cases of oral cancer were found in this house to house survey. Considering the study population as a whole, the prevalences of preleukoplakia, leukoplakia and leukokeratosis nicotina palati were 2·9 %, 4·9 % and 9·5 % respectively. Among 497 leukoplakias, 193 were associated with other oral lesions, mainly preleukoplakia and leukokeratosis nicotina palati. Table III shows the distribution of preleukoplakia, leukoplakia, and leukokeratosis nicotina palati according to sex and age groups. As in Table II the figures are expressed per 1000 villagers to facilitate comparisons between the sexes and age groups. Absolute figures for the number of persons affected by each type of lesion can be obtained by using Table III in conjunction with Table I. For all three conditions there is a predominance among females, the female : male ratios for preleukoplakia, leukoplakia, and leukokeratosis nicotina palati being 1·2 : 1, 1·5 : 1, and 2·1 : 1 respectively.

TABLE III.—*Distribution of Lesions Among Study Population. Age- and Sex-Specific Figures Per* 1000 *Villagers*

| Age group (in years) | Preleukoplakia | | Leukoplakia | | Leukokeratosis nicotina palati | |
|---|---|---|---|---|---|---|
| | Male | Female | Male | Female | Male | Female |
| 15–24 | 5·3 | 6·7 | 5·3 | 5·7 | 9·0 | 22·0 |
| 25–34 | 15·4 | 15·8 | 20·8 | 26·1 | 36·3 | 98·0 |
| 35–44 | 34·9 | 37·2 | 42·7 | 75·3 | 84·6 | 169·2 |
| 45–54 | 36·4 | 50·3 | 76·5 | 103·2 | 127·4 | 210·3 |
| 55–64 | 74·7 | 76·2 | 96·7 | 138·6 | 107·7 | 217·1 |
| 65 and over | 61·2 | 64·3 | 81·6 | 164·3 | 115·6 | 221·4 |
| All ages | 27·1 | 31·3 | 38·9 | 59·9 | 63·5 | 130·7 |

TABLE IV.—*Location of Oral Cancer, Leukoplakia and Preleukoplakia.* (*Leukokeratosis Nicotina Palati is not Included in this Table because, by Definition, Only Palatal Mucosa can be Affected*)

| Location | Oral cancer | Leukoplakia | | Preleukoplakia | |
|---|---|---|---|---|---|
| | | Number | Percentage | Number | Percentage |
| Labial mucosa | | | | | |
| Upper | — | — | — | — | — |
| Lower | — | 3 | 0·5 | 1 | 0·3 |
| Commissures | | | | | |
| Right | — | 28 | 4·7 | 22 | 5·7 |
| Left | — | 20 | 3·4 | 20 | 5·2 |
| Buccal mucosa | | | | | |
| Right | — | 57 | 9·6 | 67 | 17·3 |
| Left | — | 43 | 7·3 | 62 | 16·1 |
| Gingiva | 1 | 1 | 0·2 | — | — |
| Palate | 9 | 422 | 71·3 | 168 | 43·5 |
| Tongue | 1 | 16 | 2·7 | 46 | 11·9 |
| Floor of the mouth | — | — | — | — | — |
| Alveolar ridge | — | 2 | 0·3 | — | — |
| Total | 11 | 592 | 100·0 | 386 | 100·0 |
| Number of persons | 10 | 497 | — | 296 | — |

Examination of the data presented in Table III shows that the overall experience holds true for each separate age group, the prevalence of leukokeratosis nicotina palati being consistently greater than the prevalence of leukoplakia, and this in turn being consistently more prevalent than preleukoplakia. Also the predominance of all conditions among females holds true for all age groups. As a general rule each condition, in both sexes, shows an increasing prevalence with advancing age; the main exception to this is the peak prevalence for leukokeratosis nicotina palati occurring in the 45–54 year age group.

Table IV gives the location of oral cancer, leukoplakia and preleukoplakia. The most striking feature is the extremely high number of leukoplakias and preleukoplakias located on the palate. In addition, out of 10 oral cancer cases 9 were located to the palate; one of these patients also had cancer of the base of the tongue.

*Correlation of lesions with habits*

Table V gives the correlation between oral lesions and smoking and chewing habits. Of the 497 persons with leukoplakia, 413 (83·1 %) were also reverse smokers. When habits were correlated with the location of leukoplakias it was found, from collected data not presented in this paper, that 91·7 % of the palatal leukoplakias were in reverse smokers. Amongst the full complement of reverse smokers, 8·8 % had leukoplakia. Only 0·1 % of those persons with no habits had leukoplakia. Preleukoplakia showed correlation with tobacco habits similar to leukoplakia.

TABLE V.—*Correlation of Oral Lesions with Tobacco Habits. The Figures in Parentheses Denote the Percentages of Persons with Lesions Within Each Habit Group*

| | Number of persons | Oral cancer | Leukoplakia | Preleukoplakia | Leukokeratosis nicotina palati |
|---|---|---|---|---|---|
| Chewing habits | | | | | |
| with tobacco | 281 | — | — | 2 (0·7) | 1 (0·4) |
| without tobacco | 56 | — | — | — | — |
| Smoking habits | | | | | |
| Bidi | 587 | — | 19 (3·2) | 17 (2·9) | 3 (0·5) |
| Others | 1113 | — | 39 (3·5) | 34 (3·0) | 56 (5·0) |
| Reverse smoking | 4709 | 9 (0·2) | 413 (8·8) | 217 (4·6) | 846 (17·9) |
| Mixed habits | 803 | 1 (0·1) | 23 (2·9) | 18 (2·2) | 52 (6·5) |
| No habits | 2620 | — | 3 (0·1) | 8 (0·3) | 12 (0·5) |
| Total | 10169 | 10 (0·1) | 497 (4·9) | 296 (2·9) | 970 (9·5) |

With regard to leukokeratosis nicotina palati, 846 of the 970 persons affected (87·2 %) were reverse smokers. Taking all reverse smokers into account, 17·9 % were diagnosed as having leukokeratosis nicotina palati.

A further breakdown of the lesions is shown in Table VI, which presents the age and sex distribution of lesions in reverse smokers only. Figures are presented per 1000 reverse smokers. All lesions show a predominance among females and an increasing prevalence with increasing age. Comparison with Table III shows the extent to which these lesions are more frequent among the reverse smoking population compared to the study population as a whole.

TABLE VI.—*Distribution of Lesions Among Reverse Smokers. Age- and Sex-Specific Figures Per* 1000 *Reverse Smokers*

| Age group (in years) | Preleukoplakia | | Leukoplakia | | Leukokeratosis nicotina palati | |
|---|---|---|---|---|---|---|
| | Male | Female | Male | Female | Male | Female |
| 15–24 | 4·3 | 14·2 | 12·9 | 14·2 | 42·9 | 49·8 |
| 25–34 | 19·4 | 24·1 | 21·8 | 42·6 | 92·0 | 153·2 |
| 35–44 | 43·9 | 56·1 | 61·1 | 117·3 | 164·1 | 251·6 |
| 45–54 | 43·0 | 58·3 | 124·1 | 133·5 | 198·1 | 259·4 |
| 55–64 | 108·5 | 98·5 | 155·7 | 169·2 | 188·7 | 267·7 |
| 65 and over | 120·7 | 80·0 | 172·4 | 220·0 | 224·1 | 290·0 |
| All ages | 43·0 | 48·1 | 74·8 | 96·1 | 145·9 | 202·0 |

### *Histological findings*

*Palatal biopsies.*—Of the 381 biopsies from palatal lesions 321 were from leukoplakias, 27 from preleukoplakias and 33 from leukokeratosis nicotina palati. A characteristic feature for all three conditions was a marked hyperorthokeratosis, seen in 80 % (Fig. 5; Fig. 4 is from an individual without lesions). The hyperorthokeratosis was associated with epithelial hyperplasia in 73·1 %. Occasionally, variations were found in the pattern of hyperorthokeratosis. Fig. 6 illustrates such a case in which the granular cell layer is widely dispersed throughout the upper half of the epithelium. Other pathological features in the three conditions were the occurrence of kionoblast-like cells in the basal layer (Fig. 7), and loss of melanin pigment from the basal cells into lamina propria (Fig. 10; Fig. 9 shows the normal occurrence of melanin-containing cells in the basal layer). It was significant that very little, if any, inflammation was found in the lamina propria of the palatal biopsies.

Epithelial atypia (dysplasia) was found in 15·3 % of the leukoplakias, in 3·6 % of the preleukoplakias, and in 9·1 % of leukokeratosis nicotina palati. The changes in atypia were most marked in the basal cell area and were often associated with a delicate pointed form of the rete ridges (Fig. 10). Other examples of epithelial atypia, also associated with reverse smoking, are seen in Fig. 11 and 12.

---

EXPLANATION OF PLATES

FIG. 2.—Examples of palatal leukoplakias in individuals with the habit of reverse smoking.
FIG. 3.—An Indian female smoking chutta with the burning end inside the mouth.
FIG. 4.—Normal palatal mucosa from a 35-year-old Indian male. × 65.
FIG. 5.—Marked hyperorthokeratosis of the palatal mucosa in a 68-year-old Indian male, who has the habit of reverse smoking. × 32.
FIG. 6.—Abnormal occurrence of keratohyaline granules up to the surface in the palatal mucosa of a 55-year-old Indian female, who has the habit of reverse smoking. × 60.
FIG. 7.—Occurrence of kionoblast-like cells in the basal layer in the palatal mucosa of a 56-year-old Indian male, who has had the habit of reverse smoking. × 136.
FIG. 8.—Normal occurrence of melanin pigment in the basal cells in the palatal mucosa of a 70-year-old Indian male. × 180.
FIG. 9.—Pathological occurrence of melanin pigment in the lamina propria in the palatal mucosa of a 25-year-old Indian female, who has the habit of reverse smoking. × 205.
FIG. 10.—Epithelial atypia associated with slender rete ridges in the palatal mucosa of a 31-year-old Indian female, who has the habit of reverse smoking. × 75.
FIG. 11.—Epithelial atypia in the palatal mucosa of a 62-year-old Indian male, who has had the habit of reverse smoking. × 70.
FIG. 12.—Epithelial atypia in the palatal mucosa of a 35-year-old Indian female, who has the habit of reverse smoking. × 60.

2a

2b

2c

3

4

Pindborg, Mehta, Gupta, Daftary and Smith.

5

6

Pindborg, Mehta, Gupta, Daftary and Smith.

7

8

Pindborg, Mehta, Gupta, Daftary and Smith.

9

10

Pindborg, Mehta, Gupta, Daftary and Smith.

11

12

Pindborg, Mehta, Gupta, Daftary and Smith.

*Buccal mucosa biopsies.*—Of the 96 biopsies from the buccal mucosa lesions, 70 were from leukoplakias and 26 from preleukoplakias. The histological findings were similar to those described in the biopsies from buccal mucosa leukoplakias and preleukoplakias from the three other states in the survey (for details see Mehta *et al.*, 1969). Epithelial atypia was found in 4·3% of the leukoplakias. The preleukoplakias showed no epithelial atypia.

### DISCUSSION

There is a striking similarity between the age and sex distribution of examined individuals and of the entire rural population (Table I). This shows that the sample was representative of the district population.

The predominance of females among reverse smokers has been consistently reported from this area (Kini and Rao, 1937; Khanolkar and Suryabai, 1945; Reddy and Rao, 1957) and our observations confirm these reports. In all of the countries, except Sardinia, mentioned in the introduction reverse smoking is most often practised by females.

It is striking that so many females are addicted to the habit of reverse smoking. Of course, there are also many males who smoke in the reverse way but it is said that in Srikakulam the habit has originated among females and men have started copying them.

There are several explanations given as to why reverse smoking started and is continuing among females. First, females started smoking in the reverse way because they wanted to keep it secret from their husbands. Only a little credit can be given to this explanation as now the extent of reverse smoking among women is well known. However, it is difficult to find out whether or not a person is smoking in the reverse way if they choose not to blow out smoke for some time, as just the edge of the chutta may protrude from the lips.

Secondly, the strong winds, or splashing of water during household work, increase the chances of extinguishing the chutta if it is smoked in the conventional way. This explanation can be given some credit as it is known that the habit of reverse smoking is more common, and probably originated, among fisher-women. Quigley *et al.* (1966) give similar reasons for reverse cigarette smoking among Caribbeans.

Thirdly, the chuttas are smoked in the reverse way to prevent hot ashes falling on children and clothes, etc. A similar reason has also been given by Quigley *et al.* (1966).

Fourthly, a peculiar and interesting reason, unreported so far, is a treatment for toothache. When a person complains of toothache he is advised to smoke in the reverse way. The heat generated by reverse smoking probably produces a soothing sensation and then reverse smoking may continue as a habit.

The last, but not the least important reason given, is that the reverse smoking is due to tradition. Females see their mothers and other females smoke this way and then perhaps it appears more natural for them to start smoking in the reverse manner. It is interesting to observe that, though at all age groups there is a higher prevalence of reverse smokers among women than among men (Table II), there is a greater discrepancy in the young and old age groups than in the middle age groups. The lower figures obtained for the younger male age groups may be a result of the greater prevalence of ordinary smoking found among these groups.

Follow-up studies should reveal whether or not the lower prevalence of reverse smoking in younger age groups represents a true decline in the habit, or whether more of the population take up the habit as they grow older.

Similarly, the increased prevalence of ordinary smoking in the younger male age groups should, if continued throughout life, alter the pattern of pathological lesions associated with this habit. Follow-up studies will also reveal if the increased prevalence of chewing habits among younger females persists with increasing age and whether or not this is associated with a change in the pattern of presenting lesions.

The finding of 10 persons with oral cancer, one with two separate lesions, is difficult to assess in this type of population, for no previous similar studies have established an expected rate. However, it appears to be highly suggestive of the considerable degree of risk associated with reverse smoking when it is seen that 9 out of the 10 oral cancer cases affected the palate, and that 9 of the 10 patients were reverse smokers.

The habit of reverse smoking seems to be carrying a considerable amount of risk. Among reverse smokers roughly 25 % had some oral lesion, whereas among conventional chutta smokers only about 10 % had oral lesions (Table V). However, this does not necessarily incriminate reverse smoking in the causation of these lesions because, as shown in Table II, reverse smoking is more prevalent in women and in the older age groups whereas conventional chutta smoking is more prevalent among males and in the younger age groups. Consequently, the increased number of observed lesions in reverse smokers may be attributable, at least in part, to age or sex differences. Furthermore, it is necessary to remember that this study recorded solely the presence or absence of particular habits and gave no indication of the intensity with which they were practised. Intensity of habit indulgence would be expected to affect the number, and perhaps type, of lesions found. Such variations in habit intensity may have a sex or age basis and could account for, or mask, differences in the prevalence of lesions observed between males and females, age groups, and types of habits.

It appears that chutta, even if smoked in the conventional way, is more often associated with oral lesions than bidi smoking, and that this difference is almost totally due to leukokeratosis nicotina palati being far more prevalent among conventional chutta smokers than among bidi smokers (Table V). Not a single leukoplakia was found among individuals with chewing habits. In a similar study in the Ernakulam district of Kerala (Mehta *et al.*, 1969) leukoplakias were found among 1·8 % of chewers.

The finding of 13 individuals with leukokeratosis nicotina palati among non-smokers may require some explanation. The only explanation that can be given at present is that some individuals may have given wrong information.

The location distribution of leukoplakia and preleukoplakia shows that a large majority of these lesions were located to the palate. When correlating the tobacco habits with location of the lesion it becomes very clear that reverse smoking almost exclusively favours the palate. Similar findings have been reported by Quigley *et al.* (1966).

Indications for the premalignant nature of the lesions induced by reverse smoking are found in the frequency of epithelial atypia, 15·3 % and 9·1 % respectively, in leukoplakias and leukokeratosis nicotina palati. Ninety-eight per cent of the leukoplakias with epithelial atypia were located to the middle part of the hard palate. That area of the palate is subjected to the maximum amount of heat. Experimental studies on mice by Reddy *et al.* (1960) have suggested that heat functions as a co-carcinogen and accelerates neoplastic changes. Correlating atypias and habits, the chutta reverse smoking habit was associated with 45 atypias out of 49 (91·8 %) and four were associated with chutta smoking in the conventional manner. However, this distribution of atypia among the chutta habit groups is approximately the same as the distribution of lesions among the chutta habit groups (see Table V), so it would appear that reverse smoking does not particularly encourage the development of epithelial atypia.

Usually epithelial atypias in oral leukoplakias are associated with hyper-parakeratosis (Pindborg *et al.*, 1963), but in the present material the atypias were often associated with orthokeratosis. No satisfactory explanation for this can be given at present except that it may be connected with the prominence of the palatal site in the present material. With regard to thickness of the epithelium, it was characteristic in the present study that hyperorthokeratosis was associated with epithelial hyperplasia, whereas in our studies from other parts of India (Mehta *et al.*, 1969) hyperorthokeratosis was usually associated with an atrophic epithelium. It should be mentioned, however, that almost all the leukoplakias studied previously have been located to buccal mucosa. Palatal leukoplakias have never been studied in such a large number before.

Another unusual feature observed in the palatal biopsies was the loss of melanin from the basal cells into the lamina propria (Fig. 10). Apparently, the basal cells are so defective that they cannot keep the pigment, which then " drops " down into the connective tissue in a way very similar to that observed in oral submucous fibrosis (Pindborg and Sirsat, 1966) and in lichen planus (C. J. Smith, 1970, unpublished material).

Other interesting findings which we cannot explain at present are the occurrence of kionoblast-like cells in the basal layer of the diseased epithelium.

All the oral lesions diagnosed during the survey are now being subjected to a follow-up study.

The research conducted for this paper was supported in whole by funds from the National Institutes of Health, U.S. Public Health Service, under P.L. 480 research agreement No. 644,322.

The authors wish to express their profound appreciation to the dentists in the examining teams: Doctors R. B. Bhonsle, S. K. Choksi, V. V. Dandekar (+), Y. Mehta, V. K. Pitkar, P. N. Sinor, N. C. Shah, B. C. Shroff, P. S. Turner and S. Upadhyay.

The project is greatly indebted to Polaroid Land Corporation for an invaluable supply of cameras and films.

### REFERENCES

KHANOLKAR, V. R. AND SURYABAI, B.—(1945) *Archs Path.*, **40**, 351.

KINI, M. G. AND RAO, S. K. V.—(1937) *Indian med. Gaz.*, **72**, 677.

MEHTA, F. S., PINDBORG, J. J., GUPTA, P. C. AND DAFTARY, D. K.—(1969) *Cancer, N.Y.*, **24**, 832.

PINDBORG, J. J., BARMES, D. E. AND ROED-PETERSEN, B.—(1968) *Cancer, N.Y.*, **22**, 379.

PINDBORG, J. J., RENSTRUP, G., POULSEN, H. E. AND SILVERMAN, S.—(1963) *Acta odont. scand.*, **21**, 407.

PINDBORG, J. J. AND SIRSAT, S. M.—(1966) *Oral Surg.*, **22**, 764.
QUIGLEY, L. F. JR., SHKLAR, G. AND COBB, C. M.—(1966) *J. Am. dent. Ass.*, **72**, 867.
REDDY, D. G. AND RAO, V. K.—(1957) *Indian J. med. Sci.*, **11**, 791.
REDDY, D. G., REDDY, D. B. AND RAO, P. R.—(1960) *Cancer, N.Y.*, **13**, 263.